# Psychometrics evaluation of the university student engagement inventory in online learning among Arab students

**DOI:** 10.1186/s12912-023-01318-5

**Published:** 2023-05-09

**Authors:** Hamid Sharif-Nia, João Marôco, Pardis Rahmatpour, Nassim Ghahrani, Fatima Muhammad Ibrahim, Maryam Mohammad Ibrahim, Omolhoda Kaveh

**Affiliations:** 1 Education Development Center, Mazandaran University of Medical Sciences, Sari, Iran; 2 Department of Nursing, Amol Faculty of Nursing and Midwifery, Mazandaran University of Medical Sciences, Sari, Iran; 3 William James Centre for Research ISPA – Instituto Universitário, Lisboa, Portugal; 4 FLU Pedagogy, Nord University, Bodø, Norway; 5 School of Nursing and Midwifery, Alborz University of Medical Sciences, Karaj, Iran; 6 Reproductive Health Department, school of Nursing and Midwifery, Tehran university of Medical Sciences, International Campus, Tehran, Iran; 7 Department of Nursing, Sari Faculty of Nursing and Midwifery, Mazandaran University of Medical Sciences, Sari, Iran

**Keywords:** Student Engagement, University students, Online learning, Distance learning, Psychometric

## Abstract

**Aim:**

Student’ engagement is a predictor of various educational outcomes, and it is a key factor in perceived learning. This study aims to investigate the psychometric properties of University Student Engagement Inventory (USEI) among students of Arab universities.

**Methods:**

In this cross-sectional methodological study 525 Arab university students participated. Data was collected from December 2020 to January 2021. The confirmatory factor analysis used for construct validity, reliability and Invariance analysis for Sex were evaluated.

**Results:**

Confirmatory factor analysis indices confirmed the good model fit to the data (CFI_*scl*_=0.977, NFI_*scl*_=0.974, TLI_*scl*_=0.972, SRMR = 0.036, RMSEA_*scl*_=0.111, n = 525). All tested models showed strong invariance of the USEI between male and females. There was also evidence of convergent (AVE > 0.7 for all the scales) and discriminant validity (HTMT > 0.75 for all scales). Reliability evidence for the USEI measures in the sample of Arabic students was high (α_ordinal_ and ω above 0.86).

**Conclusion:**

The results of this study support the validity and reliability of the USEI with 15 items and 3 factors and demonstrate the importance of students’ engagement in the learning process, academic progress, and self-directed learning.

**Supplementary Information:**

The online version contains supplementary material available at 10.1186/s12912-023-01318-5.

## Introduction

Students’ learning experience and their engagement in the educational process is an important and challenging concept in educational systems due to its nature and complexity [1–3]. The concept of student engagement is defined as student involvement or commitment, which is a multifaceted and enigmatic meta construct [4]. Some researchers distinguish engagement into social engagement, academic engagement and intellectual engagement, behavioral and emotional engagement [5]. Regarding the different methods of engagement, the latter conceptualization displays three basic elements: behavioral engagement that is manifested through actions that may lead to specific observable outcomes, such as acquiring skills, focusing in the classroom, completing assignments and tasks; cognitive engagement (commitment), meaningful involvement of thought and intelligence processes such as relating ideas learned in the classroom to everyday life, applying educational and self-regulatory strategies and emotional engagement (investment) that means involvement of emotions, values and beliefs and the emergence of positive reactions to the learning environment, teachers and peers and the emergence of emotions such as passion, interest and a sense of belonging, optimism, self-confidence, tension and stress in the classroom [6–8].

Student’ engagement is a predictor of various educational outcomes, such as academic achievement, student satisfaction and dropout[9, 10]; In addition, it is a key factor in perceived learning [11]. Accordingly, this issue has attracted the attention of researchers, policy makers and planners in the field of education [12]. A study by Sengsouliya et al. showed that inclusive personal motivation, peers, professors, the university environment and family are predictors of academic engagement [13]. Numerous studies have shown that good engagement is associated with positive outcomes such as reduced length of study, high academic self-efficacy, self-motivation, and greater prosperity. Lack of engagement leads to boredom, elevated levels of stress and reduced interaction among learners [14–16]. In most studies related to students’ engagement, researchers emphasized the role of creating an organizational culture and a formal framework for student engagement through maximum communication, learner interaction and encouragement of collaborative learning, as well as the development of a supportive interaction network in the teacher-student and peer relationship and teacher feedback [13, 16, 17].

In recent years, when traditional learning and teaching are no longer an option [18], online learning has become the main style of learning due to its prominent role among all those who desire to learn, and in order that it’s many advantages, including creating learning opportunities for people in any place, a large number of persons can participate in online learning [19]. Studies show that despite the positive effects and the necessity of students’ participation in online learning from one side and addresses the challenges and providing solutions such as “affective expression (Sense of belonging in the course, Forming distinct impressions of classmates, Online communication as a medium for social interaction), open communication (Feeling comfortable talking/conversing through the online medium, Feeling comfortable participating/interacting in course discussions), and group cohesion (Feeling comfortable disagreeing with other course participants while maintaining a sense of trust in them, Feeling comfortable that your point of view is acknowledged by classmates)” as learners engage actively in online learning on the other hand; the participation of learners in online education is not ideal and sufficient, and there are still issues and dilemmas in this field [2, 3, 19, 20].

One of the ways to improve the educational quality of students is to examine the strengths and weaknesses and find a way to achieve the desired educational result. Therefore, there is a need for a tool to study student engagement and thereby take appropriate action to improve the quality of online education by increasing learner engagement. In addition, the education system needs specific tools to assess the achievement of desired educational goals [21].

Various scales were developed in this field. One of these is the Student Engagement Questionnaire (SEQ) developed by Kember & Leung (2009). This questionnaire has been approved to assess the learning processes of teaching in universities and provide feedback to teachers and institutions among Spanish students [22]. The University Student Engagement Inventory (USEI) is another scale developed by Marôco et al. It is based on the concept of interaction as a multidimensional structure, including cognitive, behavioral, and emotional engagement in response to a national survey of student engagement and lack of good psychometric properties. The USEI instrument, which was designed by Marôco et al. consists of three subscales and fifteen items[21]. The cognitive factor in the process of students’ engagement means students’ desire to learn new knowledge and solve their problems [23], and it’s one of the most important factors in self-regulation learning [24], and academic progress [25]. As stated before, these factors are important in achieving the desired and effective outcomes of learning [8]. The behavioral factor means students’ engagement in observable behaviors and functions [26], that is, the students’ visible behaviors that demonstrate their cognitive learning [23]. Emotional engagement refers to the students’ sense of belonging and understanding of value, attitudes, interests and interaction with others in the classroom, which motivates the students to perform their academic tasks [25], and this can directly and indirectly lead to students’ active learning, increasing their internal motivation and more engaging in the classroom [27].

Findings from recent studies evaluating the intercultural validity of USEI instruments in different countries indicate weak measurement variability between countries [23, 28]. Considering the need for a valid criterion for evaluating the engagement of university students in online learning conditions and the lack of valid tools in Arab countries, as well as the conditions governing the country’s education system [1, 29], this study aims to investigate the psychometric properties of USEI among students of Arab universities. We hypothesize that the Arab version of the USEI, used during online education, has good evidence of validity related to the internal structure (Construct, Convergent and Discriminant validity and reliability; as well as invariance for gender and degree of study).

## Methods

### Study design

This study utilized a cross-sectional methodological design.

### Participants and data collection

There were 525 Arab university students participated in this cross-sectional methodological study. Mean age was 26.6 (SD = 6.6). Most of the student were enrolled in a BSc degree (73%), 21% in a MSc and 5.5% on PhD/Doctorate degree (0.5% did not answer). The minimum sample size to perform a robust CFA analysis using DWLS/WLSMV with ordinary or binary is recommended to be ≥ 200–500 [30].

The items of USEI was created via Google form and sent to students using the online social App (Telegram, What’s App) and email from December 2020 to January 2021. To be included in the study, respondents had to be university students who (1) had been taking online classes and (2) were willing to be part of this study. Sample selection was based on convenience sampling.

### Instrument

The USEI used in this study after obtaining permission form Dr. Joao Marôco. This scale consists of 15 items in three subscales, scored on a 5-point Likert-type scale from 1 (never) to 5 (always). Also, it has a reversed scoring method was used for one negative question (item 6). Since the students are studied in international universities so they were fluent in English and the original version of the scale was sent to them.

### Data analysis

Descriptive statistics [mean, mode, standard deviation (SD), percentiles, Skewness (sk) and kurtosis (ku)] was well as frequency histograms were used to evaluate the USEI item’s psychometric properties using the *skimr* library [31] for the R statistical system [32]. Absolute values of sk and ku below three and seven, respectively were indicative of non-severe departure for the normal distribution required for items’ sensitivity and use on structural equation modeling [33, 34].

Sources of evidence related to the internal structure (construct related validity and reliability) of the USEI in an UAE students’ sample were gathered by means of Confirmatory Factor Analysis (CFA) and derived statistics. CFA was performed on the polychoric correlation matrix, given the ordinal nature of the items and non-severe departure from the normal distribution of subjacent latent variables, using the Diagonally Weighted Least Squares (DWLS) estimator implemented in the *lavaan* package [35]. The usual goodness of fit indices Comparative fit index (CFI), Tucker-Lewis index (TLI), Standardized Root Mean Square Residual (SRMR), and Root Mean Square Error of Approximation (RMSEA) were used. CFI and TLI above 0.95, as well as SRMR and RMSEA below 0.08 were indicative of very good model fit [36]. Since the polychoric matrix was used, the scaled versions of these indices, as provided by lavaan, were used.

Cronbach’s ordinal α, and McDonald’s ordinal ω were selected to assess reliability of the first order and second order USEI dimensions. Evidence for Convergent validity was gathered with Fornell and Larcker (1981) Average Variance Extracted (AVE). According to these authors AVE larger than 0.5 is evidence of convergent validity [37]. Evidence of discriminant validity between first order constructs was assessed with the criterion of AVE for two factors larger than the squared correlation between the factors; and the heterotrait-monotrait ratio of correlations (HTMT) below 0.9 is indicative of discriminant validity evidence [38] were used to probe discriminant validity of the USEI dimensions. The *semTools* package [39] was used to estimate AVE and HTMT. AVE above 0.5 was considered evidence of convergent validity [34, 37] Evidence of good internal consistency reliability were assume for a and ω above 0.7 [34],

Finally, invariance for the USEI measurement model was assessed by comparing a series of nested models ranging from no restrictions to the measurement model between groups (configural invariance), equal factor loadings (metric or week invariance), equal intercepts/thresholds (strong or scalar invariance), equal factor means (strong means invariance); and equal residuals variance (strict invariance). Invariance analysis for Sex (Female vs. Male) was performed using the *equaltestMI* package [40] with robust maximum likelihood estimation. Invariance between nested models was assumed for non-significant Δχ^2^ between two consecutive nested models, or absolute ΔCFI smaller than 0.01 [41] and ΔRMSEA smaller than 0.02 [42].

### Ethical consideration

The protocol of this study was approved by the ethic committee of Mazandaran University of Medical Sciences IR.MAZUMS.REC.1400.218. The study aims; number of items, time to complete the survey, the researcher’s affiliation and email for queries, and the ethical code of study were inserted on the first page of the online questionnaire. Participants were informed that their participation was voluntary and that their responses would be published anonymously as group data. Participants do not view the online questionnaire items until they agree to participate and click on the “next button”. In fact, they completed the online informed consent form by clicking.

## Results

### Items’ Distributional Properties

The distributional properties of the English version of the USEI are displayed in Table 1. All points of the items were selected with mean values around the center of the 5-point response options. Absolute values of Sk and Ku were below 1 confirming that no severe deviations from the normal distribution were observed. The psychometric sensitivity of the 15 USEI items were demonstrated.

**Table 1:** Distributional properties of the of the USEI in the Study sample (n = 525). Item 6 was reversed before analysis

Item	Mean	SD	Min	P25	P50	P75	Max	Sk	Ku	Histogram
It1	3.545	1.032	1	3	4	4	5	-0.244	-0.715	▁▃▇▇▅
It2	3.764	1.036	1	3	4	5	5	-0.329	-0.951	▁▃▇▇▇
It3	3.695	1.046	1	3	4	5	5	-0.378	-0.655	▁▃▇▇▇
It4	3.577	1.045	1	3	4	4	5	-0.395	-0.438	▁▃▇▇▅
It5	3.640	1.078	1	3	4	5	5	-0.381	-0.629	▁▃▇▇▇
It6r	3.290	1.091	1	3	3	4	5	-0.256	-0.581	▂▅▇▇▃
It7	3.482	1.073	1	3	4	4	5	-0.286	-0.621	▁▃▇▇▅
It8	3.653	1.089	1	3	4	5	5	-0.488	-0.446	▁▂▇▇▆
It9	3.577	1.056	1	3	4	4	5	-0.310	-0.629	▁▃▇▇▆
It10	3.518	1.096	1	3	4	4	5	-0.398	-0.557	▁▃▆▇▅
It11	3.630	1.021	1	3	4	4	5	-0.354	-0.624	▁▃▆▇▅
It12	3.552	1.075	1	3	4	4	5	-0.329	-0.660	▁▃▇▇▆
It13	3.690	1.014	1	3	4	4	5	-0.395	-0.502	▁▂▇▇▆
It14	3.669	0.985	1	3	4	4	5	-0.366	-0.585	▁▂▆▇▅
It15	3.596	1.017	1	3	4	4	5	-0.359	-0.541	▁▃▆▇▅

### Validity evidence based on Internal structure and measurement invariance

**Fig. 1:**
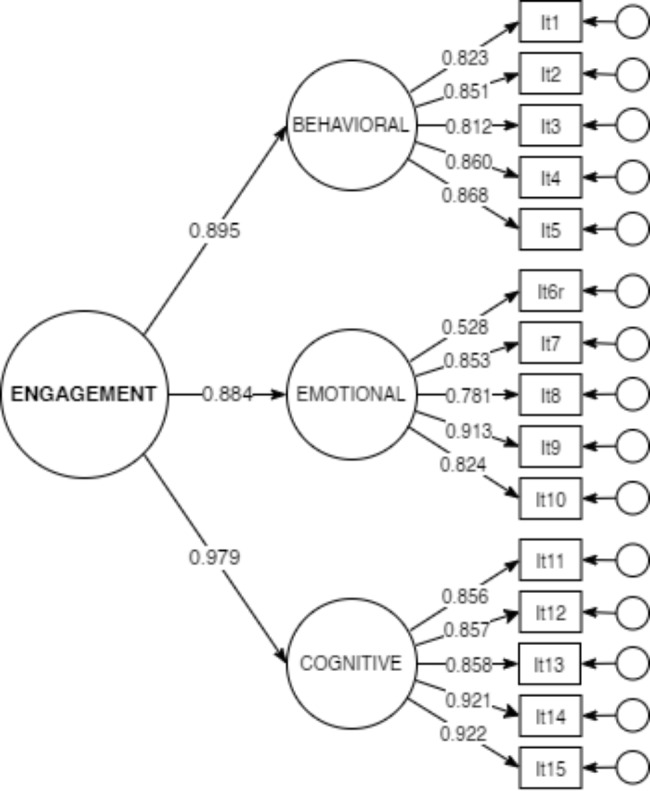
CFA model of USEI among Arab university students

The CFA analysis of the tri-factorial USEI structure showed a good fit to the data both for the first order tri-factor model, as well as for the Engagement as a second order construct (CFI_*scl*_=0.977, NFI_*scl*_=0.974, TLI_*scl*_=0.972, SRMR = 0.036, RMSEA_*scl*_=0.111). No errors’ correlations based on modification indices were introduced to improve the fit. Standardized first order and second order factor loadings are shown in Fig. 1. All loadings were statistically significant for *p* < 0.001.

Measurement invariance for sex was obtained by increased constrains on the engagement model (Configural invariance), fixed factor loadings between male and females (metric invariance), fixed factor loadings plus intercepts (scalar invariance) and fixed loading, intercepts and means (means invariance). The combination of Δχ^2^ non-significant, ΔCFI < 0.01 and ΔRMSEA < 0.02 for all tested models showed strong invariance of the USEI between male and females (see Table 2).

**Table 2:** USEI’s multi group invariance analysis for sex

Model	DF	χ2	Δχ2	ΔDF	p	CFI	RMSEA	ΔCFI	ΔRMSEA
Configural	174	551.068	-	-	-	0.938	0.091	-	-
Metric	186	570.492	19.424	12	0.079	0.937	0.089	0.001	0.002
Scalar	198	599.844	29.353	12	0.003	0.934	0.088	0.003	0.001
Means	201	603.764	3.920	3	0.270	0.934	0.087	0.000	0.001

Standardized factor loadings for the of the USEI (CFI_*scl*_=0.977, NFI_*scl*_=0.974, TLI_*scl*_=0.972, SRMR = 0.036, RMSEA_*scl*_=0.111, n = 525). Item 6 was reversed before analysis.

### Convergent and discriminant validity evidence

Convergent validity was assessed by the Average Variance Extracted (AVE). AVE was larger than 0.5 for both Behavioral (AVE = 0.711), Emotional (AVE = 0.717) and Cognitive (AVE = 0.780) engagement attesting the convergent validity of all the first order constructs. According to the Fornell & Larcker criterion [37] there was no discriminant validity between the cognitive engagement and the Emotional and Behavioral engagements- However, according to the HTMT more liberal criterion discriminant validity was observed between the three engagement constructs (See Table 3). These results confirm our research hypothesis one regarding the validity of the internal structure of the USEI.

**Table 3:** Convergent and Discriminant validity evidence by the Fornell & Larcker (1982) (a) and the HTMT (Henseler et al. 2015, 38) (b) criterion

Dimension	Behavioral	Emotional	Cognitive
(a) AVE (main diagonal) and squared correlation between first order factors			
Behavioral	0.711		
Emotional	0.611	0.717	
Cognitive	0.768	0.749	0.78
(b) HTMT			
Behavioral	1		
Emotional	0.758	1	
Cognitive	0.870	0.854	1

(a) AVE (main diagonal) and squared correlation (b) HTMT between first order factors

### Evidence of reliability

Evidence of the reliability of first order Behavioral, Emotional and Cognitive engagement dimensions was assessed with α_ordinal_ and ω. ω_L1_ was used for the second order engagement factor. All values were quite larger than 0.7 (see Table 4) indicative of reliability evidence for the USEI measures in the sample of Arabic students confirming our hypothesis two.

**Table 4:** Evidence of Reliability for the USEI measures

Statistic	Behavioral	Emotional	Cognitive
α_ordinal_	0.924	0.880	0.944
ω	0.902	0.866	0.925
	Engagement		
ω_L1_	0.917		

## Discussion

In recent years, E-learning has been implemented as a strategy against the probable stopping of the routine face-to-face educational activities [43, 44] and motivated teachers to use new teaching methods in order to increase students’ interest in the course topics [45]. In this regard, what seems critically significant is the students’ ability and motivation in independent and spontaneous learning [46, 47]. In other words, in E-learning, students should be able to actively engage through metacognitive skills, self-directed learning and self-regulation [46, 48, 49]. Thus, applying new teaching methods versus the traditional methods and lectures can increase the students’ cognitive engagement and desirable and effective learning outcomes [50]. Therefore, considering the importance of this matter; in the present study, the researchers investigated the psychometric properties of the University Student Engagement Inventory (USEI) instrument in UAE students (Al Ain University, Al Ain Campus, Abu Dhabi University, United Arab Emirates University and Higher Colleges of Technology).

The 5-item cognitive subscale got the lowest variance in the study of Marôco et al. [21]. However, in the present study, the regression coefficient was used that was reported higher than other factors (γ = 0.979) in explaining the concept of students’ engagement, which was also the most important factor explaining the concept of engagement in the Persian version [23]. The highest factor loadings of this tool are related to this subscale’s items. These items include item 15 in the cognitive factor (the factor loading = 0.922) as “Student’s effort in integrating the subjects from different disciplines into their general knowledge” and then item 14 in the cognitive factor (the factor loading = 0.921) as “Student’s effort in applying the acquired knowledge in solving the problem.“ The research cases indicated that integrating the subjects into the scientific concepts can highly help teachers and students to adapt education to their needs more easily [51]. Moreover, problem solving is important in improving students’ cognitive level [52].

In the study of Marôco et al., the 5-item behavioral subscale has the highest variance among other factors [21]. But in this study, γ = 0.895 regression coefficient was the second factor explaining the concept of students’ engagement. learners’ behavioral patterns lead to improved learning, the effective organization of knowledge, and strengthening the students’ awareness [53].

In the study of Marôco et al., according to the variance, the 5- item emotional factor is the second most effective factor in the concept known as the students’ engagement [21]. But in the present study, with a regression coefficient of γ = 0.884, it is the third explanatory factor of this concept. The result of the research reported that the students’ emotional engagement is more important than their cognitive engagement in obtaining favorable learning outcomes [54]. However, the lowest factor load of this instrument is related to item 6 with the emotional coefficient (the factor loading = 0.528),which is an inverse item referring to the student’s lack of success in the classroom, and this was also seen in the psychometrics of the Persian version of this instrument [23]. The effect of this reversed item was consistent also in other studies of the psychometric properties of the USEI in different countries [26].

As the results of this study revealed, the Arabic version of USEI displayed acceptable internal consistency and construct reliability, and satisfactory convergent and divergent validity. The results of the USEI assessment among the students also reported the USEI a valid and reliable assessment for studying the students’ engagement worldwide [28].

Therefore, in the Arabic version of the inventory, just like the original version, by identifying three cognitive, behavioral, and emotional subscales, it was determined that in addition to the cognitive factors, the behavioral and emotional factors are also very important in the students’ engagement in the classroom and achieving the desired learning outcomes. This research gathered evidence for the validity of the internal structure and reliability of the USEI when applied to an Arab student population. Cultural differences in the value of education between genders in Arabic cultures reflect the need for the analysis of sex invariance. The Arab version of the USEI was invariant between males and females. Good evidence related to the internal structure (factorial, convergent, discriminant validity and reliability) the invariance shows that USEI can be used to produce valid and reliable data on engagement for both sexes.

### Strengths and limitations

Considering the importance of the role of cognitive, emotional and behavioral engagement of students in the classroom on their satisfaction and academic progress, it is necessary for educators to become more familiar with the types of students’ engagement, in order to create a useful educational experience for them. So this scale is useful for the researchers and university administrators to accurately measure the engagement of Arab students, but this study was conducted on UAE students, due to cultural differences, it may be limited to conduct this study in other countries, and finally that may limit the generalizability of findings; as well as The self-report method of the survey may have led to some errors. But one of the important limitations of this research, has been the study of Arab students’ engagement in online classes and online learning, which can be investigated in future studies of students’ engagement in other types of e-learning such as blended learning.

## Conclusion

Good evidence related to the internal structure (factorial, convergent, discriminant validity and reliability) the invariance shows that USEI can be used to produce valid and reliable data on engagement for both sexes.

## Electronic supplementary material

Below is the link to the electronic supplementary material.

Supplementary Material 1

## Data Availability

Due to the privacy of the research participants, the data generated during the current study are not publicly available but are available from the corresponding author upon reasonable request.
