# Supplementary material for: Psychometrics evaluation of the university student engagement inventory in online learning among Arab students
Source: BMC Nurs. 2023 May 9;22:158. doi: 10.1186/s12912-023-01318-5 (PMC10169459; doi:10.1186/s12912-023-01318-5)
Supplement: Supplementary file 1 — Supplementary Material 1 [file 12912_2023_1318_MOESM1_ESM.docx]

**Psychometrics Evaluation of the University Student Engagement Inventory in Online Learning among Arab Students**

Hamid Sharif-Nia^1,2^, João Marôco^3^, Pardis Rahmatpour^4^, Nassim Ghahrani^1^,Fatima Muhammad Ibrahim^5^, Maryam Muhammad Ibrahim ^5^, [Omolhoda Kaveh](https://www.ncbi.nlm.nih.gov/pubmed/?term=Kaveh%20O%5BAuthor%5D&cauthor=true&cauthor_uid=31592042)*^6^

1. Educational Development Center, Mazandaran University of Medical Sciences, Sari, Iran
2. Department of Nursing, Amol Faculty of Nursing and Midwifery, Mazandaran University of Medical Sciences, Sari, Iran
3. William James Centre for Research ISPA – Instituto Universitário, Lisboa, Portugal & FLU Pedagogy. Nord University. Bodø. Norway.
4. School of Nursing and Midwifery, Alborz University of Medical Sciences, Tehran, Iran
5. PhD Reproductive Health Department, school of Nursing and Midwifery, Tehran university of Medical Sciences, International Campus, Tehran, Iran
6. Department of Nursing, Sari Faculty of Nursing and Midwifery, Mazandaran University of Medical Sciences, Sari, Iran*

**Corresponding author:** [Omolhoda Kaveh](https://www.ncbi.nlm.nih.gov/pubmed/?term=Kaveh%20O%5BAuthor%5D&cauthor=true&cauthor_uid=31592042)*

School of Nursing and Midwifery Sari, Mazandaran University of Medical Sciences, Sari, Iran

**Tel: +**989101051394

**Email:** kaveh.hoda@yahoo.com
